# An observational study of innate immune responses in patients with acute appendicitis

**DOI:** 10.1038/s41598-020-73798-3

**Published:** 2020-10-15

**Authors:** Toon Peeters, Sandrina Martens, Valentino D’Onofrio, Mark H. T. Stappers, Jeroen C. H. van der Hilst, Bert Houben, Ruth Achten, Leo A. B. Joosten, Inge C. Gyssens

**Affiliations:** 1 Department of Infectious Diseases and Immunity, Jessa Hospital, 3500 Hasselt, Belgium; 2 Faculty of Medicine and Life Sciences, Hasselt University, 3500 Hasselt, Belgium; 3 Department of Internal Medicine, Radboud University Medical Center, 6525 GA Nijmegen, The Netherlands; 4 Department of Abdominal and Oncological Surgery, Jessa Hospital, 3500 Hasselt, Belgium; 5 Department of Pathology, Jessa Hospital, 3500 Hasselt, Belgium; 6 Department of Medical Genetics, Iuliu Haţieganu University of Medicine and Pharmacy, Cluj-Napoca, Romania; 7 Department of Internal Medicine AIG 463, Radboud University Medical Center, P.O. Box 9101, 6500 HB Nijmegen, The Netherlands; 8 Present Address: Department of Biosciences, Geoffrey Pope Building, University of Exeter, Stocker Road, Exeter, EX4 4QD UK; 9 Present Address: Department of Experimental Pathology, Vrije Universiteit Brussel (VUB), 1090 Brussels, Belgium

**Keywords:** Cytokines, Inflammation, Innate immunity, Gastroenterology, Risk factors

## Abstract

Acute appendicitis is a common surgical emergency worldwide. Exaggerated immune responses could be associated with appendicitis. This study aimed at characterizing immune responses towards a large variety of gut commensals and pathogens, and pattern recognition receptor (PRR) ligands, and investigating the course of systemic inflammation in a prospective cohort of acute appendicitis patients. PBMC responses of 23 patients of the cohort and 23 healthy controls were characterized more than 8 months post-surgery. Serum cytokine levels were measured in 23 patients at the time of appendicitis and after one month. CRP, WBC and percentage of neutrophils were analyzed in the total cohort of 325 patients. No differences in PBMC responses were found between patients and controls. Stronger IL-10 responses were found following complicated appendicitis. A trend towards lower IL-8 responses was shown following gangrenous appendicitis. Serum IL-10 and IL-6 were significantly elevated at presentation, and IL-6, IL-8 and TNF-α levels were higher in complicated appendicitis. Routine biomarkers could predict severity of appendicitis with high specificities, but low sensitivities. Cytokine responses in patients following acute appendicitis did not differ from healthy controls. Higher serum cytokine levels were found in acute complicated and gangrenous cases. Further research into discriminative biomarkers is warranted.

## Introduction

Acute appendicitis is one of the most common surgical emergencies worldwide^1^, with a lifetime risk estimated between 6 and 17%^2, 3^. The disease involves inflammation of the appendix, which can be divided into different types or stages. It is mostly classified as uncomplicated versus complicated appendicitis. Complicated appendicitis is defined by the presence of peritonitis, abscesses and/or perforation. Histologically, a distinction can be made between gangrenous appendicitis, which is associated with severe transmural inflammation and areas of necrosis^4^, and non-gangrenous appendicitis.

The appendix is associated with a mass of lymphatic tissue, indicating a possible role for the appendix in immune function. The enteric immune system supports biofilm formation, and compared to other regions in the gastrointestinal tract, biofilms are most prominent in the appendix. Considering these observations, and the properties and location of the appendix, the theory was introduced that the appendix functions as a safe house for commensal bacteria, protecting them from the fecal stream and allowing them to repopulate the gut after trauma^5^.

Little is understood about the etiology of acute appendicitis. Microbiological causes and infection are often suggested^6^. There is large variability in micro-organisms detected in the appendix, indicating that a single organism is unlikely the sole cause of appendicitis^7^. The focus of studies into microbial causes of appendicitis has therefore shifted towards the microbiome as a whole. The composition of microbiota in the appendix differs from other regions in the gastrointestinal tract^8^, and there is large variation between individuals. Differences in the composition of the fecal microbiome have furthermore been associated with appendicitis^9^.

Exaggerated immune responses to commensal bacteria have been associated with auto-immune diseases^10^, and an overly active immune system has also been associated with appendicitis. After stimulation of peripheral blood mononuclear cells (PBMC) with tetanus toxoid, an increase of secretion of interferon gamma (IFN-γ) in patients with gangrenous appendicitis compared to negative appendectomy controls has been observed, and interleukin (IL)-10 secretion after stimulation is increased in gangrenous compared to phlegmonous appendicitis patients^11^.

During acute appendicitis, high IL-8 levels can be observed in the appendix, the peritoneal fluid, and according to some studies in the serum of patients^12^. In contrast to healthy appendices, inflamed appendices also demonstrate an intense cellular tumor necrosis factor (TNF)-α mRNA expression in germinal centers and moderate levels of expression throughout the mucosa, while IL-2 mRNA is strongly expressed in the lamina propria and moderately in germinal centers^13^. Gangrenous appendicitis is associated with inflammatory markers in serum, consistent with a Th17 response^14^.

C-reactive protein (CRP) is a routine inflammatory biomarker which has also been used in the development of clinical scoring systems for diagnosing appendicitis, along with white blood cell count (WBC) and percentage of neutrophils^15, 16^. IL-6 can be considered as a biomarker for appendicitis as well, since high serum levels can often be associated with the condition^17^. A recent study showed a specific metabolomic and inflammatory mediator profile in pediatric acute appendicitis, where IL-6 and CRP were found among the most distinctive inflammatory markers^18^.

High grade inflammation and tissue damage appear to be a mechanism in the development of appendicitis, possibly caused by deviant immune responses. Therefore, we hypothesized that the risk of appendicitis and the severity of inflammation are dependent on the individual’s innate immune responses towards components of the gut microbiota. The main aim of this study was to characterize potential deviant immune responses to stimulation by a large panel of gut bacteria, other commensals and relevant corresponding Pattern Recognition Receptor (PRR) ligands.

A second aim was to study the course of systemic inflammation in patients by comparing cytokine levels at presentation and one month after surgery, and to identify discriminative biomarkers for clinical severity by comparing serum cytokine levels at time of presentation between complicated and uncomplicated appendicitis patients.

## Methods

### Study population and design

**Figure 1:**
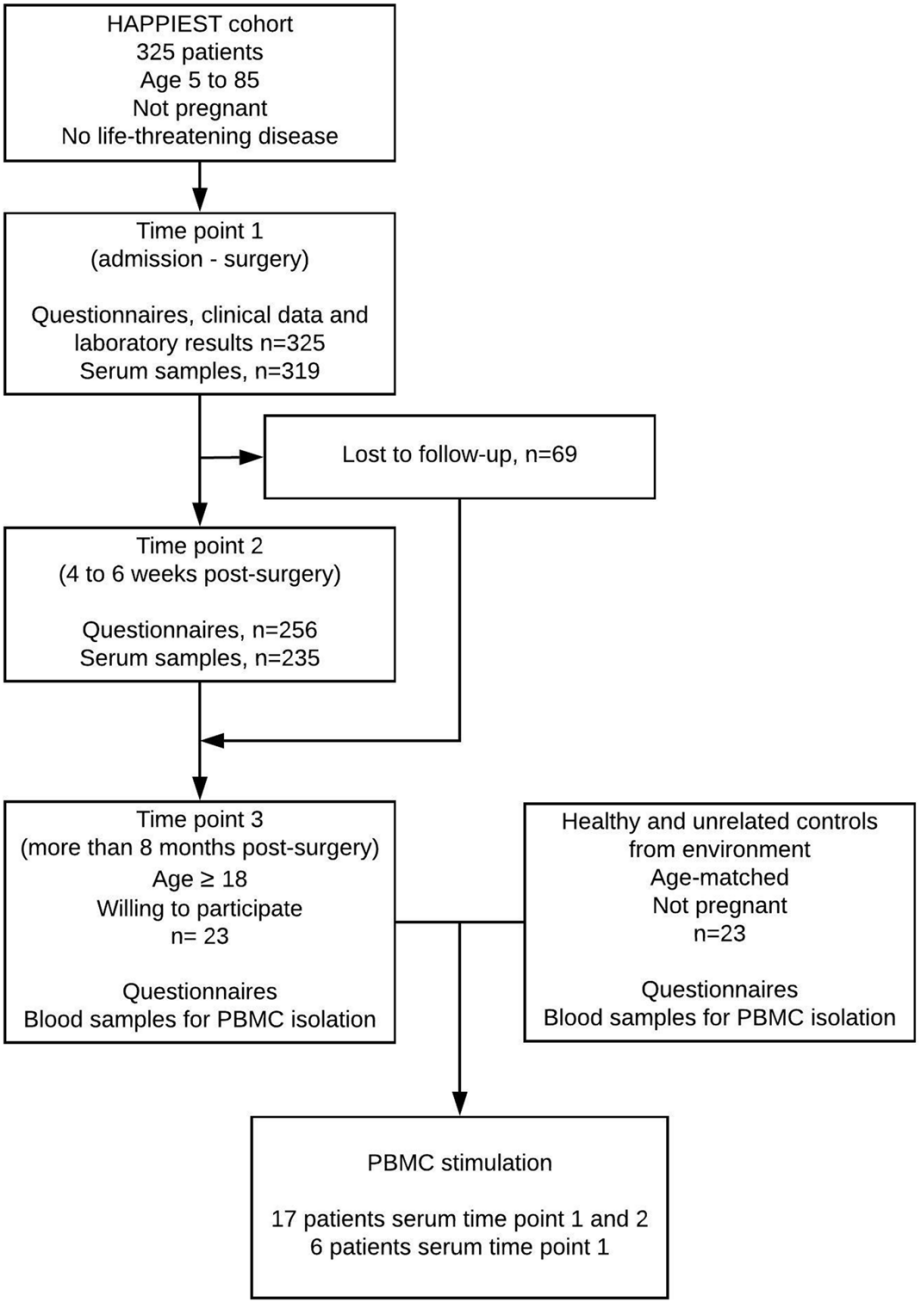
Flowchart of recruitment and sampling from patients with a history of acute appendicitis and healthy controls.

Figure 1 depicts the study design. Patients were prospectively recruited in the Hasselt Appendicitis Immunology and Environmental Study (HAPPIEST) cohort between June 2012 and October 2016. Acute appendicitis was diagnosed at the emergency department of Jessa Hospital, Hasselt, Belgium based on medical history, clinical examination, laboratory results and ultrasound and/or CT scan. Medical history, clinical data and laboratory results were recorded. At each time point, study participants filled out questionnaires. Questionnaires covered demographic data such as age, gender, dietary habits, lifestyle characteristics and environmental factors. Following removal, the appendix was sectioned by the surgeon in the operating room. A 1 cm section of the tip, the middle and the base were sent to the pathology department to confirm the diagnosis and assign histological severity (gangrenous vs non gangrenous), based on the classification by Carr, 2000^4^. Severity classification by the surgeon was based on the International Classification of Diseases (ICD)-9. Appendicitis with generalized peritonitis (540.0) or peritoneal abscess (540.1) was considered complicated, appendicitis with no mention of peritonitis or abscess (540.9) was considered uncomplicated. Analyses on severity were performed based on the pathologist’s and the surgeon’s classification, respectively.

### Sampling

Immediately before induction of anesthesia for appendectomy, 2 × 5 ml serum samples were taken (Time point 1). Four to six weeks after surgery, patients returning for a control visit donated 2 × 5 ml serum (Time point 2). Samples were aliquoted and frozen at − 80 °C.

Patients between the ages of 18 and 50 were invited to return to the hospital after more than 8 months after surgery, to guarantee full recovery, and to bring an unrelated age-matched healthy volunteer as paired control. Patients and controls were not matched for gender. A total of 23 patients (out of 158 contacted) and 23 controls were recruited in pairs. Three 10 ml EDTA blood tubes were collected from patients and controls (Time point 3). Samples were immediately transported to the laboratory for peripheral blood mononuclear cell (PBMC) isolation.

#### 2.3 PBMC isolation and stimulation.

PBMC isolation was performed on EDTA blood samples collected at Time point 3, using Ficoll density centrifugation as described previously^19^. PBMCs (0.5*10^6^) were transferred to a 96 well round bottom plates and stimulated with heat-killed commensal micro-organisms and pathogens, as well as pattern recognition receptor (PRR) agonists (Invivogen, San Diego, USA) (Supplementary Table S1) in a final volume of 200 µl. After 24 h incubation at 37 °C 5% CO2 supernatants were collected and stored at − 20 °C until further analysis.

### Cytokine and chemokine levels

Serum cytokine and chemokine levels were determined by enzyme-linked immunosorbent assays (ELISA), according to manufacturer’s instructions: IL-1β, IL-6, IL-10 and TNF-α (Ella, Proteinsimple, California, USA); Alpha-1 antitrypsin (HycultBiotech, Uden, The Netherlands); IL-1RA, MIP-1α (Quantikine, R&D systems, Minneapolis, USA); MCP-1 (Duoset ELISA, R&D systems). In supernatant, IL-1β, IL-6, IL-8, IL-10, MIP-1α and MCP-1 levels were measured (DuoSet ELISA). A number of supernatant samples were lost during analyses due to technical error or insufficient material. Supplementary Table S2 shows the number of participants per measured cytokine/chemokine and stimulus.

### Data analysis

Cytokine responses in PBMC were compared between patients and controls using Mann–Whitney U tests. Serum cytokine levels were compared between Time point 1 and 2 using Wilcoxon signed rank tests. Serum cytokine levels at the time of appendicitis were compared between uncomplicated and complicated appendicitis, and non-gangrenous and gangrenous appendicitis, using Mann–Whitney U tests. CRP, WBC and percentage of neutrophils were compared between uncomplicated and complicated, as well as non-gangrenous and gangrenous appendicitis in the total cohort matched for age and gender, using two-tailed T-tests. In order to test the diagnostic value of WBC, percentage of neutrophils and CRP as potential biomarkers for disease severity, Receiver Operating Characteristics (ROC) curves were made. Chi^2^ tests were used to compare uncomplicated and complicated, and non-gangrenous and gangrenous appendicitis for elevated clinical laboratory values. Correlations between serum cytokines levels and CRP and WBC at the time of appendicitis, as well as correlation between differences in cytokine levels between Time point 1 and 2, were analyzed using Pearson correlation. A p < 0.05 was considered statistically significant. All statistical analyses were performed using IBM SPSS statistics 25.

### Ethical statement

The study protocol was approved by the Medical Ethics Committees of Jessa Hospital and Hasselt University, Hasselt, Belgium. All experiments were performed in accordance with regulations of these institutions. The University Biobank Limburg (UBiLim) meets all national and international standards and regulations.

All patients and controls or their parents or guardians included in this study gave written informed consent before participation.

The study was registered at Clinicaltrials.gov, under identifier NCT02391675.

## Results

### Study population

Patient and control characteristics are listed in Table 1. To ensure that there was no selection of patients based on demographics, environment or lifestyle, characteristics were compared between the patient population at Time point 3 and the entire HAPPIEST population. To ensure that potential differences in immune factors between patients and controls were not a consequence of differences in demographics, environment or lifestyle, comparisons on these factors were made between both populations. There were no significant differences between patients and controls, or between the patient sample and the entire cohort, regarding demographics and exposures. Considering the potential impact of both age and gender on immune function, a comparison was made between the mean age of males and females. No significant difference was found (mean age respectively 31.50 and 33.45 years; p = 0.308). In the patient sample, there was an equal number of patients with complicated appendicitis according to ICD-9 and gangrenous appendicitis (n = 7), yet only three cases were classified as both complicated and gangrenous. In the total cohort, 96/325 (30%) of patients were classified as complicated according to ICD-9, and 103/325 (32%) were classified as gangrenous. Only 51, however, were classified as both complicated and gangrenous.

**Table 1:** Characteristics of patients with a history of appendicitis and controls.

	HAPPIEST cohort (n = 325)	HAPPIEST Patient sample (n = 23)	Healthy Controls (n = 23)	p-value^1^	p-value^2^
	n (%)	n (%)	n (%)		
Demographics					
Gender				0.767	0.546
Female	153 (47.1)	11 (47.8)	10 (43.5)		
Male	172 (52.9)	12 (52.2)	13 (56.5)		
Age, mean ± SD (Range)	32.5 ± 18.1 (5–81)	32.2 ± 6.2 (20–41)	32.6 ± 6.4 (18–43)	0.814	0.411
Marital state				1.000	0.909
Single	26 (8.4)	2 (9.1)	2 (9.1)		
Living with partner, family or in community	284 (91.6)	20 (90.9)	20 (90.9)		
Missing	15	1	1		
Living area				0.531	0.373
Rural	129 (41.5)	7 (31.8)	9 (40.9)		
Urban	182 (58.5)	15 (68.2)	13 (59.1)		
Missing	14	2	2		
Acute appendicitis, IBD					
Family history of acute appendicitis				0.082	0.521
Yes	168 (57.3)	10 (50.0)	5 (23.8)		
No	125 (42.7)	10 (50.0)	16 (76.2)		
Missing	32	3	2		
Family history of Inflammatory Bowel Disease				0.859	0.472
Yes	11 (4,4)	1 (9.1)	1 (7.1)		
No	237 (95,6)	10 (90.9)	13 (92.9)		
Don’t know	44	0	0		
Missing	33	12	9		
Complicated (ICD-9)					0.910
Yes	96 (29.5)	7 (30.4)	n.a.		
No	229 (70.5)	16 (69.6)	n.a.		
Gangrenous (pathology)					
Yes	103 (31.7)	7 (30.4)	n.a.		0.845
No	222 (68.3)	16 (69.6)	n.a.		
Time between first symptoms and presentation (hours)					
Complicated (ICD-9)					0.091
Less than 24	11 (12.0)	0 (0.0)	n.a.		
24–48	29 (31.5)	5 (71.4)	n.a.		
More than 48	52 (56.5)	2 (28.6)	n.a.		
Missing	4	0			
Uncomplicated (ICD-9)					0.752
Less than 24	61 (27.5)	5 (31.3)	n.a.		
24–48	85 (38.3)	7 (43.8)	n.a.		
More than 48	76 (34.2)	4 (25.0)	n.a.		
Missing	7	0			
Gangrenous (pathology)					0.030
Less than 24	20 (20.0)	1 (14.3)	n.a.		
24–48	37 (37.0)	6 (85.7)	n.a.		
More than 48	43 (43.0)	0 (0.0)	n.a.		
Missing	3	0			
Non-Gangrenous (pathology)					0.985
Less than 24	52 (24.3)	4 (25.0)	n.a.		
24–48	77 (36.0)	6 (37.5)	n.a.		
More than 48	85 (39.7)	6 (37.5)	n.a.		
Missing	8	0			
Exposures					
Living area in youth				0.894	0.470
Rural	159 (51.3)	10 (43.5)	10 (45.5)		
Urban	151 (48.7)	13 (56.5)	12 (54.5)		
Missing	15	0	1		
Contact with farm animals during youth				0.286	0.340
Seldom or never	181 (58.0)	11 (47.8)	14 (63.6)		
Daily to monthly	131 (42.0)	12 (52.2)	8 (36.4)		
Missing	13	0	1		
Living with pets				0.746	0.883
Yes	178 (56.7)	11 (55.0)	11 (50.0)		
No	136 (43.3)	9 (45.0)	11 (50.0)		
Missing	11	3	1		
Breastfeeding				0.102	0.537
No	87 (33.2)	5 (26.3)	9 (52.9)		
Yes	175 (66.8)	14 (73.7)	8 (47.1)		
Don't know	45	4	5		
Missing	18	0	1		
Duration				0.368	0.212
Less than 3 months	47 (39.8)	2 (25.0)	0 (0.0)		
3–6 months	38 (32.2)	5 (62.5)	4 (100.0)		
More than 6 months	33 (28.0)	1 (12.5)	0 (0.0)		
Don't know	57	6	4		
Missing	0	0	0		
Vegan/vegetarian				1.000	0.495
Yes	7 (2.2)	1 (4.5)	1 (4.5)		
No	305 (97.8)	21 (95.5)	21 (95.5)		
Missing	13	1	1		
Meat consumption				0.379	0.567
Daily	235 (79.1)	15 (75.0)	18 (90.0)		
Weekly	57 (19.2)	4 (20.0)	2 (10.0)		
Monthly	5 (1.7)	1 (5.0)	0 (0.0)		
Missing	21	1	1		
Fruit consumption				0.269	0.987
Daily	130 (41.8)	9 (40.9)	13 (61.9)		
Weekly	132 (42.4)	10 (45.5)	8 (38.1)		
Monthly	30 (9.6)	2 (9.1)	0 (0.0)		
Never	19 (6.1)	1 (4.5)	0 (0.0)		
Don't know	1	0	1		
Missing	13	1	1		
Vegetable consumption				0.550	0.771
Daily	259 (82.7)	20 (90.9)	21 (95.5)		
Weekly	48 (15.3)	2 (9.1)	1 (4.5)		
Monthly	1 (0.3)	0 (0.0)	0 (0.0)		
Never	5 (1.6)	0 (0.0)	0 (0.0)		
Missing	12	1	1		
Fiber-rich vegetable consumption				0.167	0.283
Daily	34 (11.2)	2 (9.1)	2 (9.1)		
Weekly	207 (68.1)	13 (59.1)	18 (81.8)		
Monthly	51 (16.8)	7 (31.8)	2 (9.1)		
Never	12 (3.9)	0 (0.0)	0 (0.0)		
Don't know	7	0	0		
Missing	14	1	1		
Sugar containing drink consumption				0.190	
Daily	–	12 (57.1)	15 (68.2)		
Weekly	–	4 (19.0)	5 (22.7)		
Monthly	–	4 (19.0)	0 (0.0)		
Never	–	1 (4.8)	2 (9.1)		
Don't know	–	1	0		
Missing	–	1	1		
Antibiotic use				0.101	0.281
More than once per month	1 (0.3)	0 (0.0)	0 (0.0)		
Monthly	15 (4.8)	0 (0.0)	0 (0.0)		
Seldom	250 (80.4)	15 (71.4)	20 (90.9)		
Never	45 (14.5)	6 (28.6)	2 (9.1)		
Missing	14	2	1		
Probiotic use				0.239	0.649
Daily	63 (20.2)	6 (28.6)	5 (22.7)		
Weekly	64 (20.5)	4 (19.0)	8 (36.4)		
Monthly	26 (8.3)	2 (9.5)	5 (22.7)		
Seldom	88 (28.2)	7 (33.3)	8 (36.4)		
Never	71 (22.8)	2 (9.5)	0 (0.0)		
Missing	13	2	1		
Smoke status				0.282	0.217
Current smoker	56 (22.0)	2 (9.1)	6 (27.3)		
Past smoker	61 (23.9)	9 (40.9)	8 (36.4)		
Non-smoker	132 (51.8)	11 (50.0)	8 (36.4)		
Passive	6 (2.4)	0 (0.0)	0 (0.0)		
Missing	70	1	1		

^1^ Patient sample versus controls.

^2^ HAPPIEST cohort versus patient sample.

### PBMC cytokine responses

**Figure 2:**
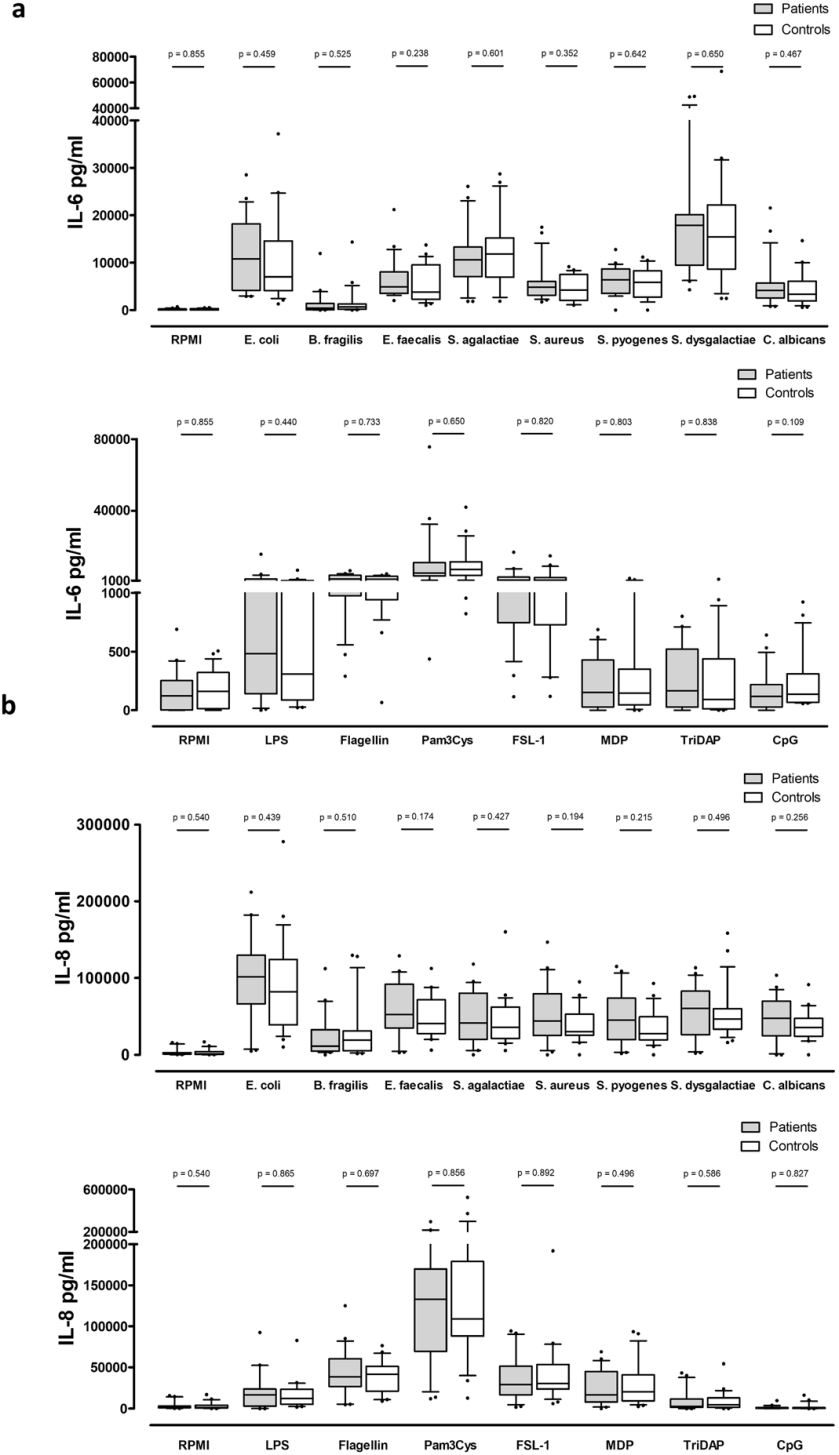

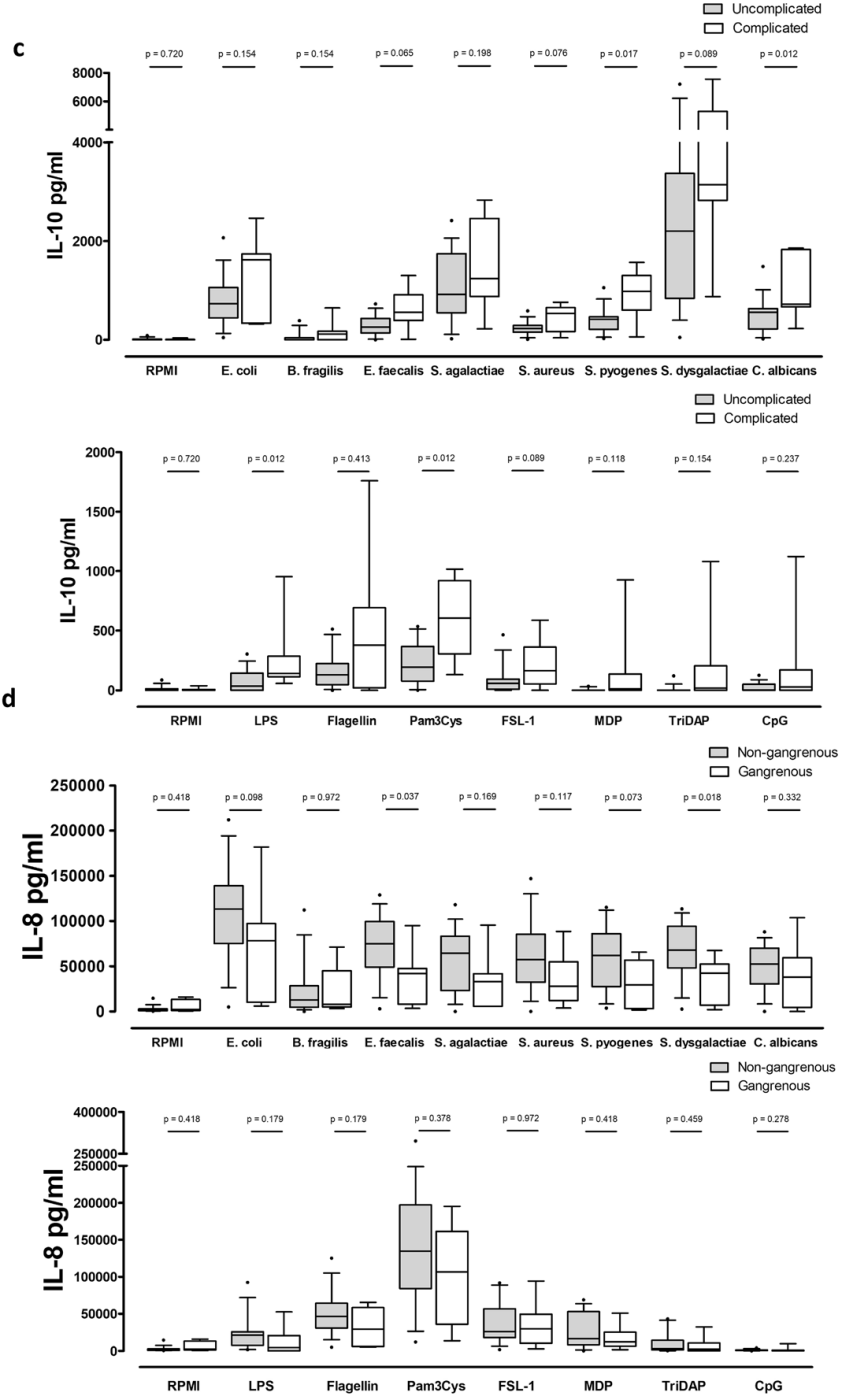
Cytokine responses towards commensal gut bacteria, pathogens and PRR agonists. (**a**, **b**) IL-6 and IL-8 responses in patients (n = 23) and controls (n = 23). (**c**) IL-10 responses in patients with a history of complicated (n = 16) and uncomplicated (n = 7) appendicitis. (**d**) IL-8 responses in patients with a history of non-gangrenous (n = 16) and gangrenous (n = 7) appendicitis. Whiskers indicate 10–90 percentile.

Trends could be observed towards higher IL-6 and IL-8 responses in patients (n = 23) compared to controls (n = 23) (Fig. 2a, b). No significant differences between patients and controls were found in IL1β, IL-6, IL-8, IL-10, MIP-1α or MCP-1 responses towards commensal gut bacteria, pathogens or PRR agonists (Fig. 2a, b, Supplementary Fig. S1).

When comparing complicated and uncomplicated appendicitis patients classified according to ICD-9, IL-10 responses towards *S. pyogenes*, *C. albicans*, LPS and Pam3Cys were significantly higher in complicated appendicitis patients (Fig. 2c). Other cytokine responses did not significantly differ between uncomplicated and complicated appendicitis patients (Supplementary Fig. S2). Significantly higher IL-6 responses towards FSL-1 (p = 0.026) and lower responses towards *S. dysgalactiae* (p = 0.026) were shown in patients with a history of gangrenous appendicitis (Supplementary Fig. S3). A clear trend towards lower IL-8 responses was shown in patients with a history of gangrenous appendicitis (Fig. 2d).

### Serum cytokine levels and other inflammatory markers

**Figure 3:**
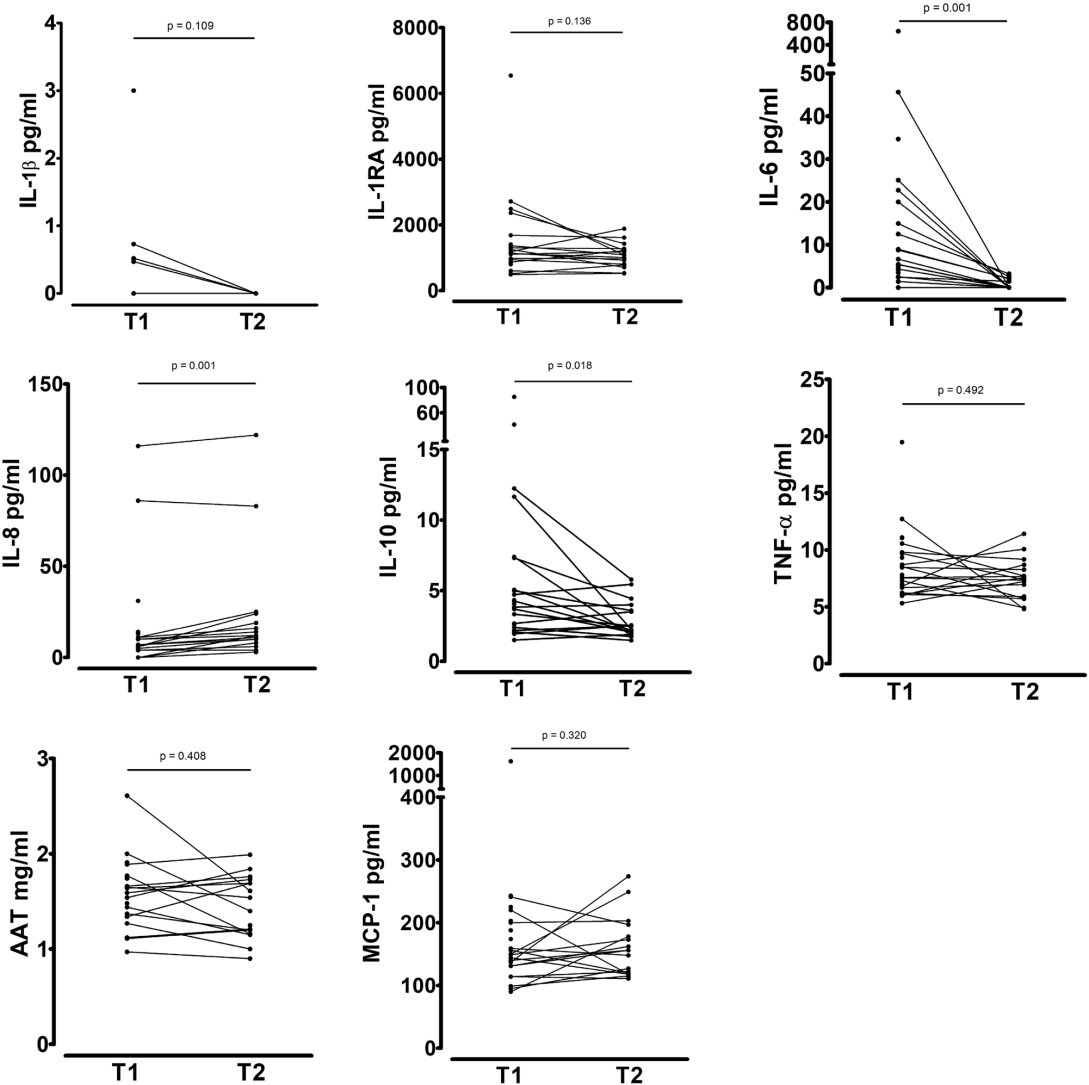
Serum cytokine concentrations in patients at the time of appendicitis and after one month of follow-up. T1 = Time point one, time of appendicitis (n = 23), T2 = Time point 2, one month of follow-up (n = 17).

From the sample of 23 patients, five had not presented for the control visit (Time point 2). One patient was suffering from sinusitis and was removed from this analysis. Serum IL-6 and IL-10 were significantly elevated during acute appendicitis, whereas IL-8 was significantly lower during appendicitis (Fig. 3). The decrease in IL-10 at the control visit was correlated with the decrease in IL-6 (p < 0.001), IL-1Ra (p = 0.026) and MCP-1 (p = 0.019). The decrease in IL-6 was also correlated with a decrease in IL-1Ra (p = 0.023) and MCP-1 (p = 0.020). There was also a correlation in the decrease in MCP-1 and IL-1Ra (p = 0.028).

**Figure 4:**
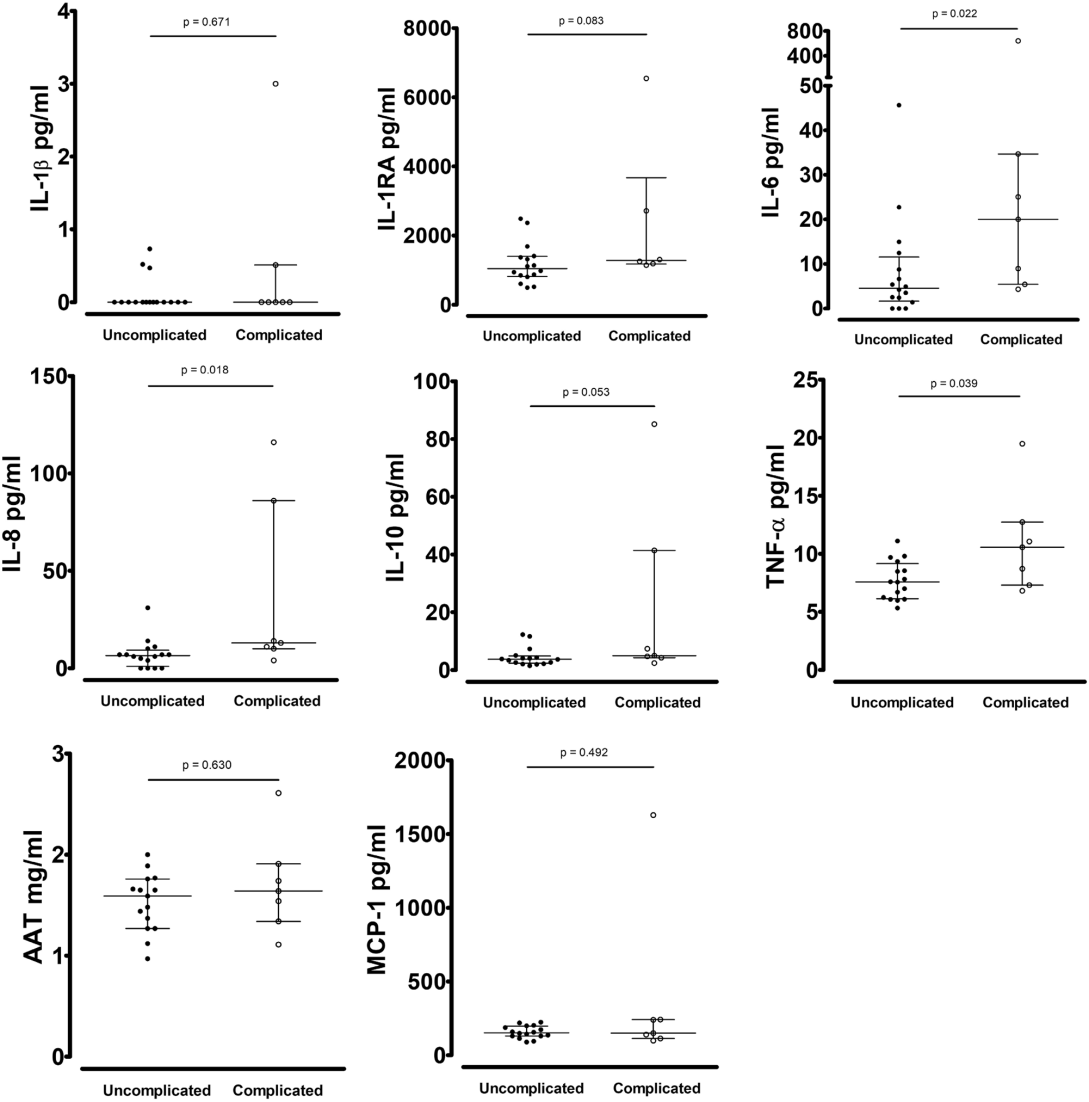
Serum cytokine levels in patients with uncomplicated (n = 16) and complicated (n = 7) appendicitis.

At presentation, serum IL-6, IL-8 and TNF-α levels were significantly higher in complicated versus uncomplicated appendicitis patients (Fig. 4). Patients with gangrenous appendicitis had significantly higher serum IL-10 levels (p = 0.018). CRP at presentation was correlated with IL-1RA (p = 0.003), IL-8 (p = 0.042), IL-10 (p = 0.033), TNF-α (p = 0.001) and AAT (p = 0.003). No correlations with WBC could be observed. MIP-1α levels were below the detection limit.

**Figure 5:**
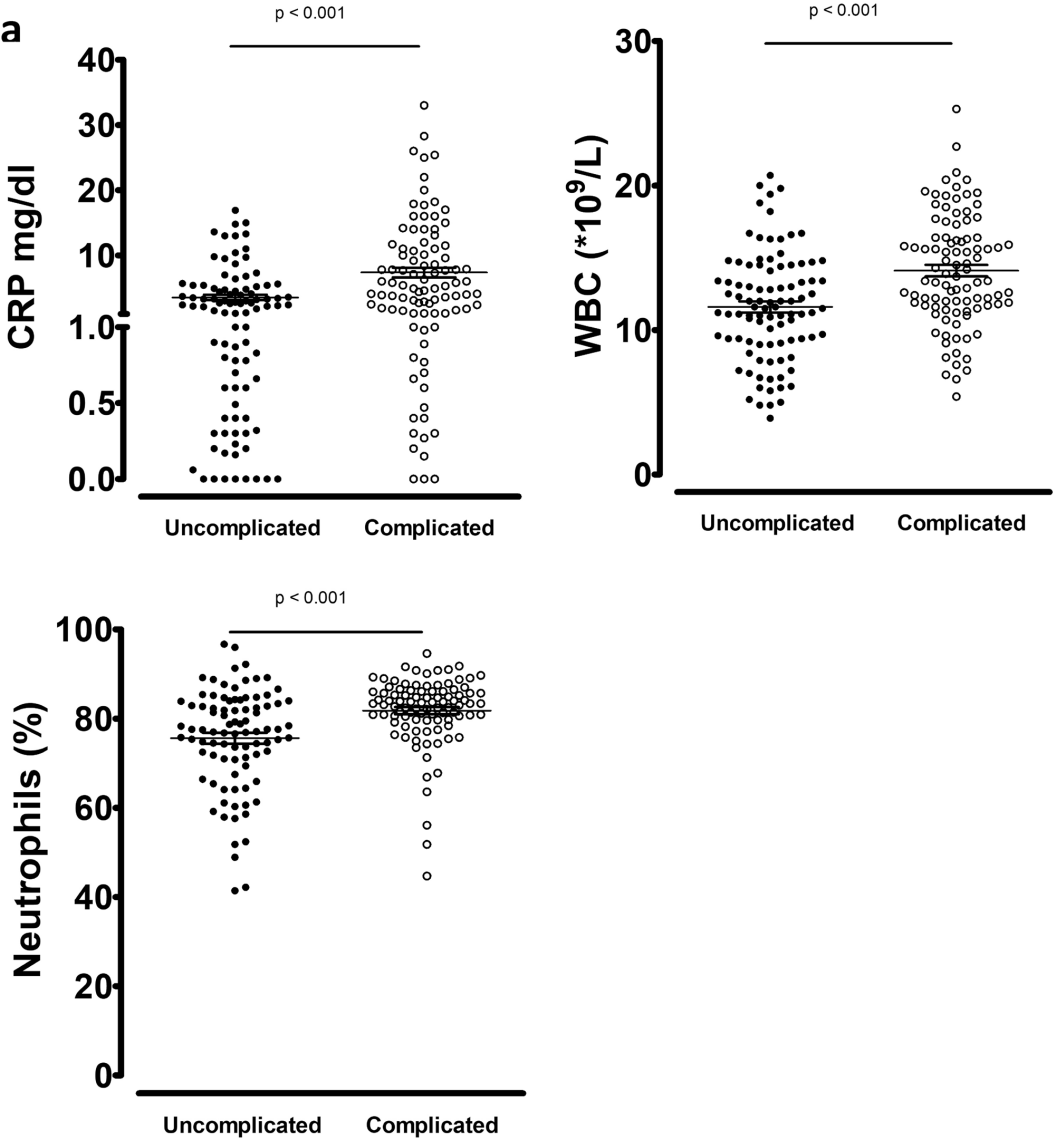

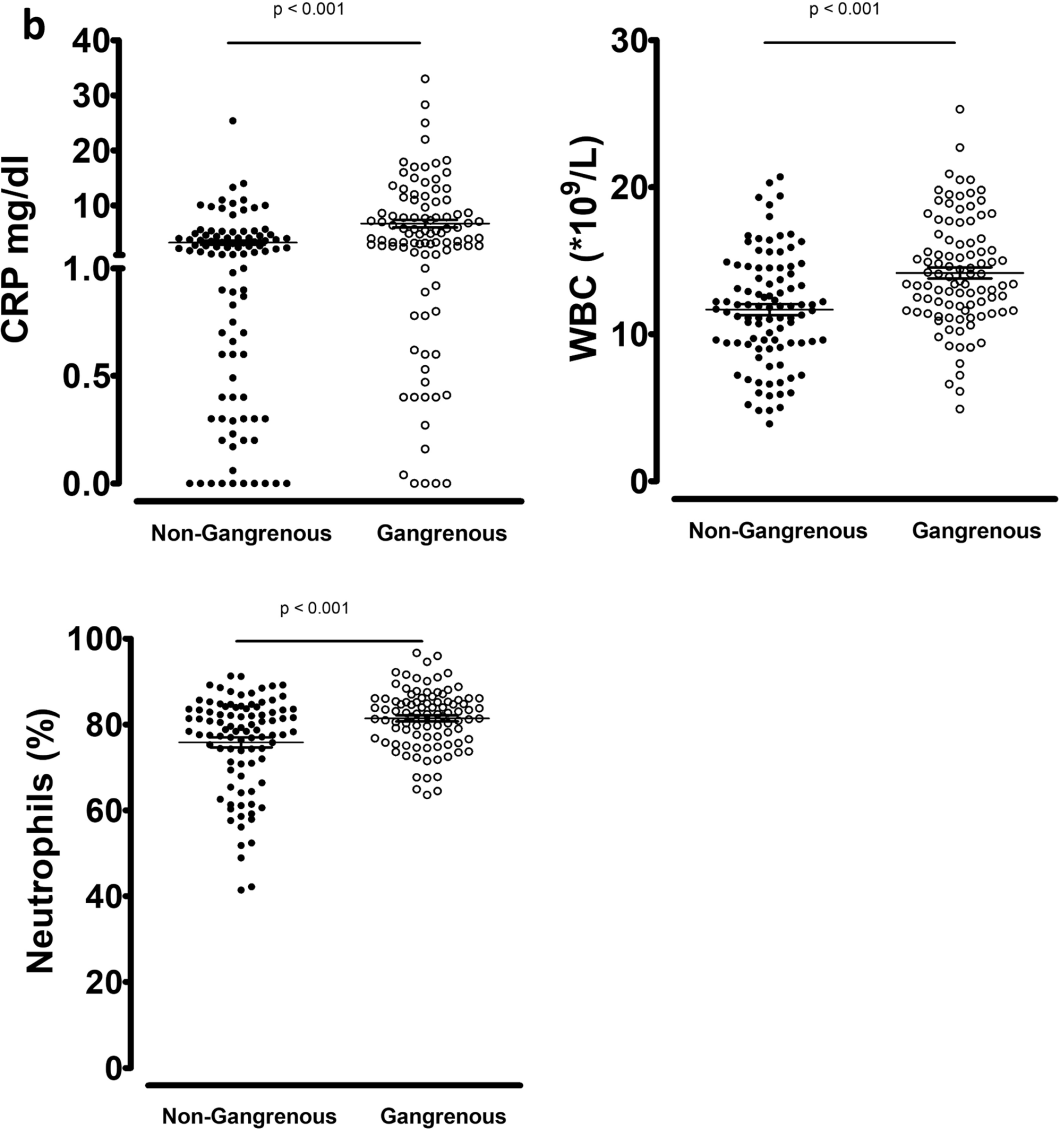
Routine biomarkers according to severity in the total cohort matched for age and gender. (**a**) Uncomplicated (n = 95) versus Complicated (n = 95) according to ICD-9. (**b**) Non-Gangrenous (n = 97) versus Gangrenous (n = 97) according to pathology. Bars indicate mean with SEM.

In the analysis of the total cohort matched for age and gender, CRP, WBC and percentage of neutrophils were significantly higher in complicated appendicitis and in gangrenous appendicitis (p < 0.001, Fig. 5). Supplementary Tables S3 and S4 show the number of patients with normal and elevated routine biomarkers at presentation according to severity, as well as the positive and negative predictive values, and Area Under Curve (AUC) derived from ROC curves. The threshold of 10*10^9^ WBC/L could predict uncomplicated appendicitis with specificity of 0.884 (sensitivity 0.242), and the threshold of 70% neutrophils could predict uncomplicated appendicitis with specificity of 0.937 (sensitivity 0.236). The upper normal limit of CRP at 0.5 mg/dl, set in accordance with hospital laboratory standards, could predict uncomplicated appendicitis with a specificity of 0.853 (sensitivity 0.326). WBC, percentage of neutrophils and CRP could predict non-gangrenous appendicitis with specificity of 0.866 (sensitivity 0.268), 0.941 (sensitivity 0.237) and 0.897 (sensitivity 0.340), respectively. Patients with a history of symptoms for more than 24 h before presenting at the hospital, more frequently had elevated CRP (p = 0.001) and WBC (p = 0.010). Combining these biomarkers and delay offered only minimal improvement in predicting the severity of appendicitis (data not shown).

## Discussion

This study illustrates involvement of the innate immune system in acute appendicitis, and its severity. There was a clear trend towards higher IL-10 responses in PBMCs from patients with a history of complicated compared to uncomplicated appendicitis, and responses towards a number of specific stimuli were significantly elevated. This is in line with increased IL-10 after stimulation of PBMCs in gangrenous appendicitis in earlier research^11^. IL-6 polymorphisms have previously been associated with severity of appendicitis^20^, stronger IL-6 responses in patients with a history of gangrenous appendicitis could provide a functional explanation. Remarkably, trends towards lower IL-8 responses in patients with a history of gangrenous appendicitis were observed. The potential differences according to severity of the disease, however, do not necessarily indicate a causal relationship. Although time point 3 was specifically selected to provide enough time for potential effects of appendicitis or surgery to disappear, disease outcome may still have long term effects on immune function, lasting longer than the 8 month period between acute appendicitis and experiments in this study.

No significant differences in PBMC responses were observed between patients and controls. However, since trends were observed towards higher IL-6 and especially IL-8 responses in patients, the possibility of a role for these two cytokines cannot completely be eliminated. Large variation in immune responses in patients and controls may have influenced all analyses. Further research in a larger sample is therefore warranted. Another explanation for these findings could be that deviant immune responses associated with acute appendicitis are confined to the appendix itself or the enteric immune system^21^.

A strength of this study is the selection of control individuals for the stimulation experiments from the environment of patients in order to eliminate potential environmental influences; in addition, the time point of the stimulation experiments was late enough to guarantee full recovery and avoid effects of trained immunity in the majority of patients. These effects are pronounced after 3 months, but wane after 6 months to 1 year^22^. There was no selection for the stimulation experiments other than the fact that active and healthy former patients were willing to return to the hospital with a non-related healthy age-matched individual as paired control. An additional strength is the use of classification both by the surgeon and the pathologist. As seen in the results in this study, there was no large overlap between patients with complicated appendicitis and gangrenous appendicitis. Using both classification systems allowed for easier comparison with the literature^11, 14^ and provided a broader image of different pathological processes in acute appendicitis. Using a large variety of relevant stimuli and measuring multiple cytokine and chemokine concentrations also contributed to a higher level of certainty that innate immune responses in patients with a history of appendicitis were not significantly different from healthy control individuals in this population.

Elevated IL-6 and IL-10 levels in serum of acute appendicitis patients are in line with previous findings^23^, although the decrease in serum levels of these markers one month following appendicitis has, to our knowledge, not been documented. Since no other serum markers assessed in this study were significantly elevated, these cytokines are likely the most important as drivers of acute appendicitis.

There was no correlation of IL-6 levels with CRP or WBC measurement at presentation, IL-10 was correlated with CRP. A possible explanation can be a difference in the kinetics of these biomarkers during the inflammatory process. Correlations between IL-10, IL-6, IL-1Ra and MCP-1 indicate that a combination of these factors could be of importance in appendicitis.

High serum levels of IL-8 in patients compared to healthy controls were not found in this study. However, all inflammatory cytokines examined in this study tended to be higher in complicated versus uncomplicated appendicitis. Serum IL-10 was significantly higher in gangrenous appendicitis. Differences in results between these classification systems possibly indicate the different systemic consequences of pathologic processes in the appendix versus peritonitis and periappendicular abscesses. AAT and MCP-1 showed no discriminative power as biomarkers for severity, which to our knowledge has not been described in the literature.

Analyses on the entire cohort showed the ability to predict uncomplicated and non-gangrenous appendicitis using CRP, WBC or percentage of neutrophils with high specificity, indicating that low values could be of use as markers for less severe appendicitis. Apart from their role in scores for the diagnosis of acute appendicitis, their possible use in predicting disease severity has been the focus of other studies as well^24–26^. The possibility to differentiate between uncomplicated and complicated, non-gangrenous and gangrenous appendicitis at presentation using routine biomarkers is interesting considering the discussion on antibiotic therapy as an alternative to surgery in cases of uncomplicated appendicitis^27, 28^. High WBC, CRP and percentage neutrophils could indicate a higher possibility of severe appendicitis. However, low values did not rule this out, and basing a decision for surgery on these biomarkers is not without risk. Furthermore, CRP levels and WBC were associated with delay, which is known to be the main risk factor of more severe appendicitis^29^. As there are correlations between biomarkers and delay, combining these factors did not improve prediction of severity.

In conclusion, ex-vivo experiments of PBMC stimulation showed little to no evidence of innate immune dysfunction as an inherent characteristic of patients with a history of acute appendicitis, although differences in responses between patients with a history of complicated and uncomplicated appendicitis do show a possible link. Systemic inflammation and correlations between serum levels of cytokines warrant further research into the possibility of combining markers that can be valuable in the determining the severity of appendicitis at presentation.

## Data availability

The datasets generated during and/or analysed during the current study are available from the corresponding author on reasonable request.

## Supplementary information

Supplementary Information.
