# Supplementary material for: An observational study of innate immune responses in patients with acute appendicitis
Source: Sci Rep. 2020 Oct 15;10:17352. doi: 10.1038/s41598-020-73798-3 (PMC7562899; doi:10.1038/s41598-020-73798-3)
Supplement: Supplementary file 1 — Supplementary Information. [file 41598_2020_73798_MOESM1_ESM.pdf]

An observational study of innate immune responses in patients with acute appendicitis.

Toon Peeters[1, 2, 3, 4], Sandrina Martens[1,2], Valentino D'Onofrio[1,2,3,4], Mark H.T. Stappers[3], Jeroen CH van der Hilst[1,2], Bert Houben[5], Ruth Achten[6], Leo A.B. Joosten[3,4,7], Inge C. Gyssens[1,2,3,4*]

1. Department of Infectious Diseases & Immunity, Jessa Hospital, 3500 Hasselt, Belgium

2. Faculty of Medicine and Life Sciences, Hasselt University, 3500 Hasselt, Belgium

3. Department of Internal Medicine, Radboud University Medical Center, 6525 GA Nijmegen, The Netherlands

4. Radboudumc Center for Infectious Diseases (RCI), Radboud University Medical Center, 6525 GA Nijmegen, The Netherlands

5. Department of Abdominal and Oncological Surgery, Jessa Hospital, 3500 Hasselt, Belgium

6. Department of Pathology, Jessa Hospital, 3500 Hasselt, Belgium

7. Department of Medical Genetics, Iuliu Haţieganu University of Medicine and Pharmacy, Cluj-Napoca, Romania.

Corresponding author

Prof. Dr. I.C. Gyssens

Radboud University Medical Center

Dept. of Internal Medicine AIG 463

P.O. Box 9101

6500 HB Nijmegen, the Netherlands

+31 24 361 1899

Inge.gyssens@radboudumc.nl

Current affiliations

Mark H.T. Stappers: Department of Biosciences, Geoffrey Pope Building, University of Exeter, Stocker Road, Exeter, EX4 4QD, UK.

Sandrina Martens: Department of Experimental Pathology, Vrije Universiteit Brussel (VUB), 1090 Brussels, Belgium.

Supplementary Table S1. Stimuli (micro-organisms and ligands) used in PBMC stimulation

Heat-killed bacteria/fungi	Concentration	PRR Agonists	Concentration
Bacteroides fragilis ATCC 25285	$1x10^6$ cfu/ml	Pam3Cys (TLR2/1)	10 µg/ml
Escherichia coli ATCC 25922	$1x10^6$ cfu/ml	FSL-1 (TLR2/6)	1 µg/ml
Enterococcus faecalis ATCC 29212	$1x10^6$ cfu/ml	LPS (*E. coli*) (TLR4)	1 ng/ml
Staphylococcus aureus ATCC 29213	$1x10^6$ cfu/ml	Flagellin (TLR5)	1 µg/ml
Streptococcus pyogenes ATCC 19615	$1x10^6$ cfu/ml	CpG (TLR9)	10 µg/ml
Streptococcus agalactiae ATCC 12386	$1x10^6$ cfu/ml	MDP (NOD2)	10 µg/ml
Streptococcus dysgalactiae CCUG 36637	$1x10^6$ cfu/ml	Tri-DAP (NOD1)	10 µg/ml
Candida albicans UC820	$1x10^6$ cfu/ml		

Supplementary Table S2. Number of subjects per stimulus and measured cytokine

Stimulus	IL-1β	IL-6	IL-8	IL-10	MCP-1	MIP-1α
RPMI	46	45	45	46	44	44
Heat Killed bacteria/fungi						
B. fragilis	45	45	45	46		
E. coli	46	44	44	46	42	43
E. faecalis	45	45	43	46	40	44
S. aureus	46	45	43	46	40	44
S. pyogenes	45	45	43	44		
S. agalactiae	42	45	45	46		
S. dysgalactiae	43	45	45	46		
C. albicans	45	45	45	46		
PRR agonists						
Pam3Cys	45	45	45	46		
FSL-1	43	45	45	46		
LPS	45	45	43	46		
Flagellin	44	45	42	46		
CpG	45	43	43	45		
MDP	44	45	45	46		
Tri-DAP	44	45	45	46		

Supplementary Table S3. Routine biomarkers at presentation in uncomplicated and complicated appendicitis

	Uncomplicated (n=95) n (%)	Complicated (n=95) n (%)	p-value	PPV	NPV	AUC*
CRP			0.004	0.559	0.689	0.670
Normal	31 (32.6)	14 (14.7)				
Elevated	64 (67.4)	81 (85.3)				
WBC			0.023	0.538	0.676	0.680
Normal	23 (24.2)	11 (11.6)				
Elevated	72 (75.8)	84 (88.4)				
Percentage neutrophils			0.001	0.567	0.778	0.688
Normal	21 (23.6)	6 (6.3)				
Elevated	68 (76.4)	89 (93.7)				
Missing	6	0				

* AUC for state uncomplicated, lower values indicate positive test

PPV: Positive predictive value; NPV: Negative predictive value

Supplementary table 4. Routine biomarkers at presentation in non-gangrenous and gangrenous appendicitis

	Non-gangrenous (n=97)	Gangrenous (n=97)	p-value	PPV	NPV	AUC
	n (%)	n (%)				
CRP			< 0.001	0.576	0.767	0.673
Normal	33 (34.0)	10 (10.3)				
Elevated	64 (66.0)	87 (89.7)				
WBC			0.020	0.542	0.667	0.681
Normal	26 (26.8)	13 (13.4)				
Elevated	71 (73.2)	84 (86.6)				
Percentage neutrophils			0.001	0.572	0.786	0.640
Normal	22 (23.7)	6 (6.3)				
Elevated	71 (76.3)	95 (93.7)				
Missing	4	2				

* AUC for state non-gangrenous, lower values indicate positive test

PPV: Positive predictive value; NPV: Negative predictive value

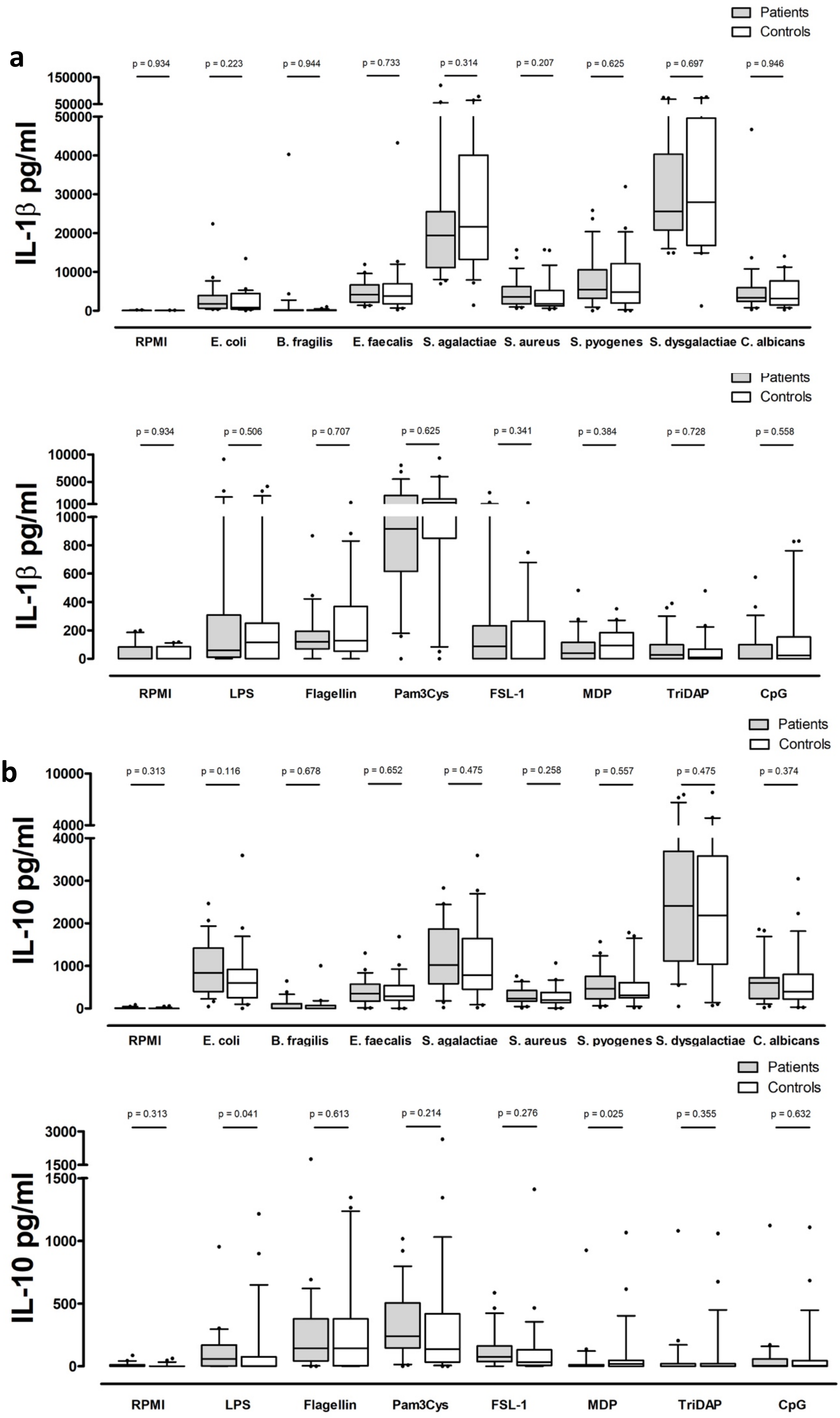
a
b
Patients
Controls
IL-1β pg/ml
IL-10 pg/ml
RPMI
E. coli
B. fragilis
E. faecalis
S. agalactiae
S. aureus
S. pyogenes
S. dysgalactiae
C. albicans
LPS
Flagellin
Pam3Cys
FSL-1
MDP
TriDAP
CpG
p = 0.934
p = 0.223
p = 0.944
p = 0.733
p = 0.314
p = 0.207
p = 0.625
p = 0.697
p = 0.946
p = 0.934
p = 0.506
p = 0.707
p = 0.625
p = 0.341
p = 0.384
p = 0.728
p = 0.558
p = 0.313
p = 0.116
p = 0.678
p = 0.652
p = 0.475
p = 0.258
p = 0.557
p = 0.475
p = 0.374
p = 0.313
p = 0.041
p = 0.613
p = 0.214
p = 0.276
p = 0.025
p = 0.355
p = 0.632

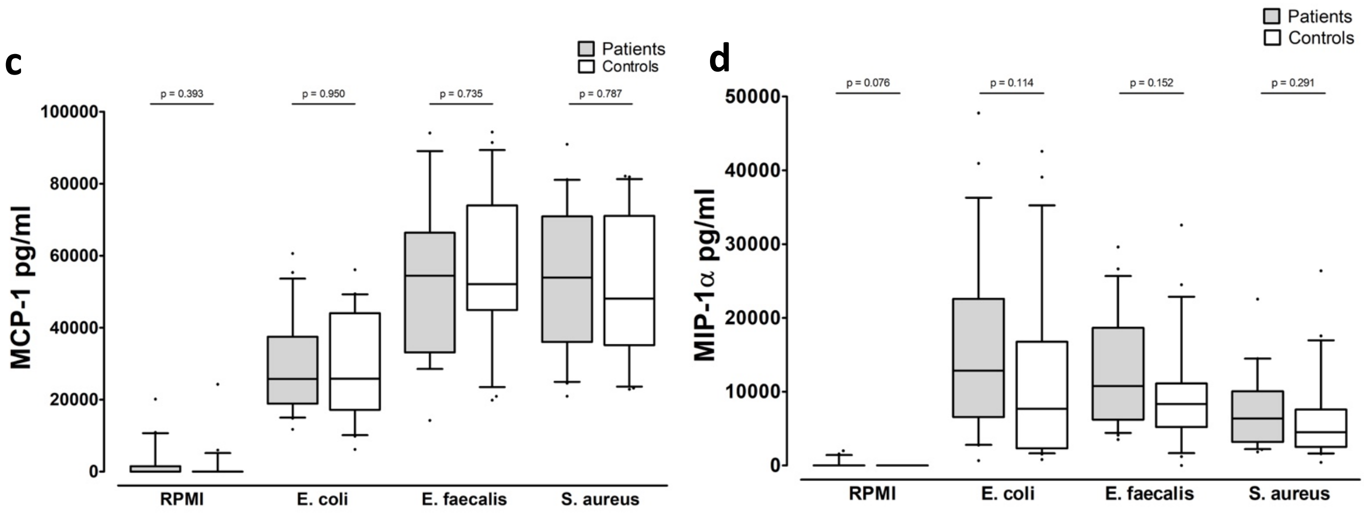
c
Patients
Controls
p = 0.393
p = 0.950
p = 0.735
p = 0.787
MCP-1 pg/ml
100000
80000
60000
40000
20000
0
RPMI
E. coli
E. faecalis
S. aureus
d
Patients
Controls
p = 0.076
p = 0.114
p = 0.152
p = 0.291
MIP-1α pg/ml
50000
40000
30000
20000
10000
0
RPMI
E. coli
E. faecalis
S. aureus

Supplementary Figure S1. Cytokine responses towards commensal gut bacteria, pathogens and PRR agonists in patients (n=23) and controls (n=23). (a) IL-1β responses. (b) IL-10 responses. (c) MCP-1 responses. (d) MIP-1α responses. Whiskers indicate 10-90 percentile.

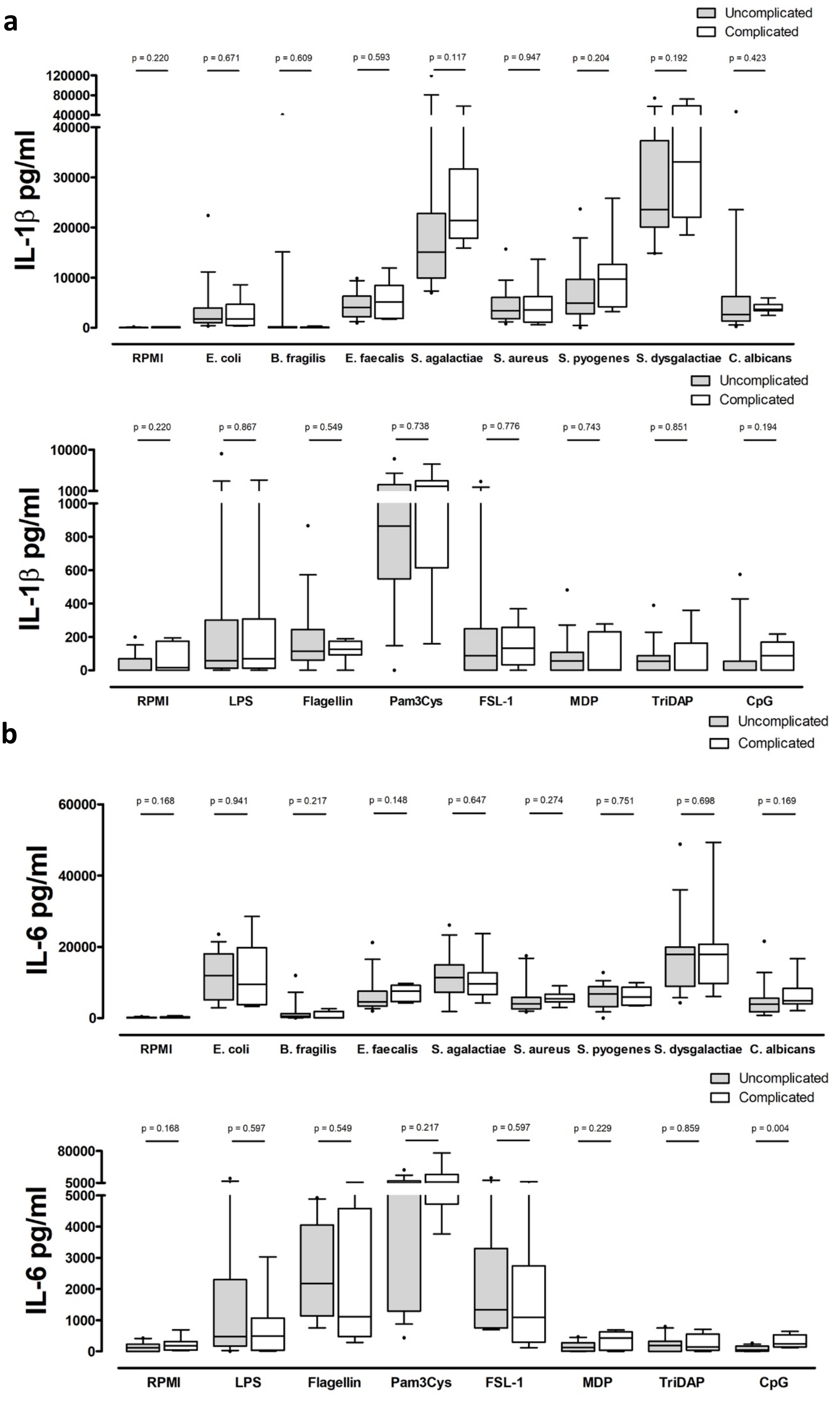
a
Uncomplicated
Complicated
IL-1β pg/ml
p = 0.220
p = 0.671
p = 0.609
p = 0.593
p = 0.117
p = 0.947
p = 0.204
p = 0.192
p = 0.423
RPMI
E. coli
B. fragilis
E. faecalis
S. agalactiae
S. aureus
S. pyogenes
S. dysgalactiae
C. albicans
p = 0.220
p = 0.867
p = 0.549
p = 0.738
p = 0.776
p = 0.743
p = 0.851
p = 0.194
RPMI
LPS
Flagellin
Pam3Cys
FSL-1
MDP
TriDAP
CpG
b
IL-6 pg/ml
p = 0.168
p = 0.941
p = 0.217
p = 0.148
p = 0.647
p = 0.274
p = 0.751
p = 0.698
p = 0.169
p = 0.168
p = 0.597
p = 0.549
p = 0.217
p = 0.597
p = 0.229
p = 0.859
p = 0.004

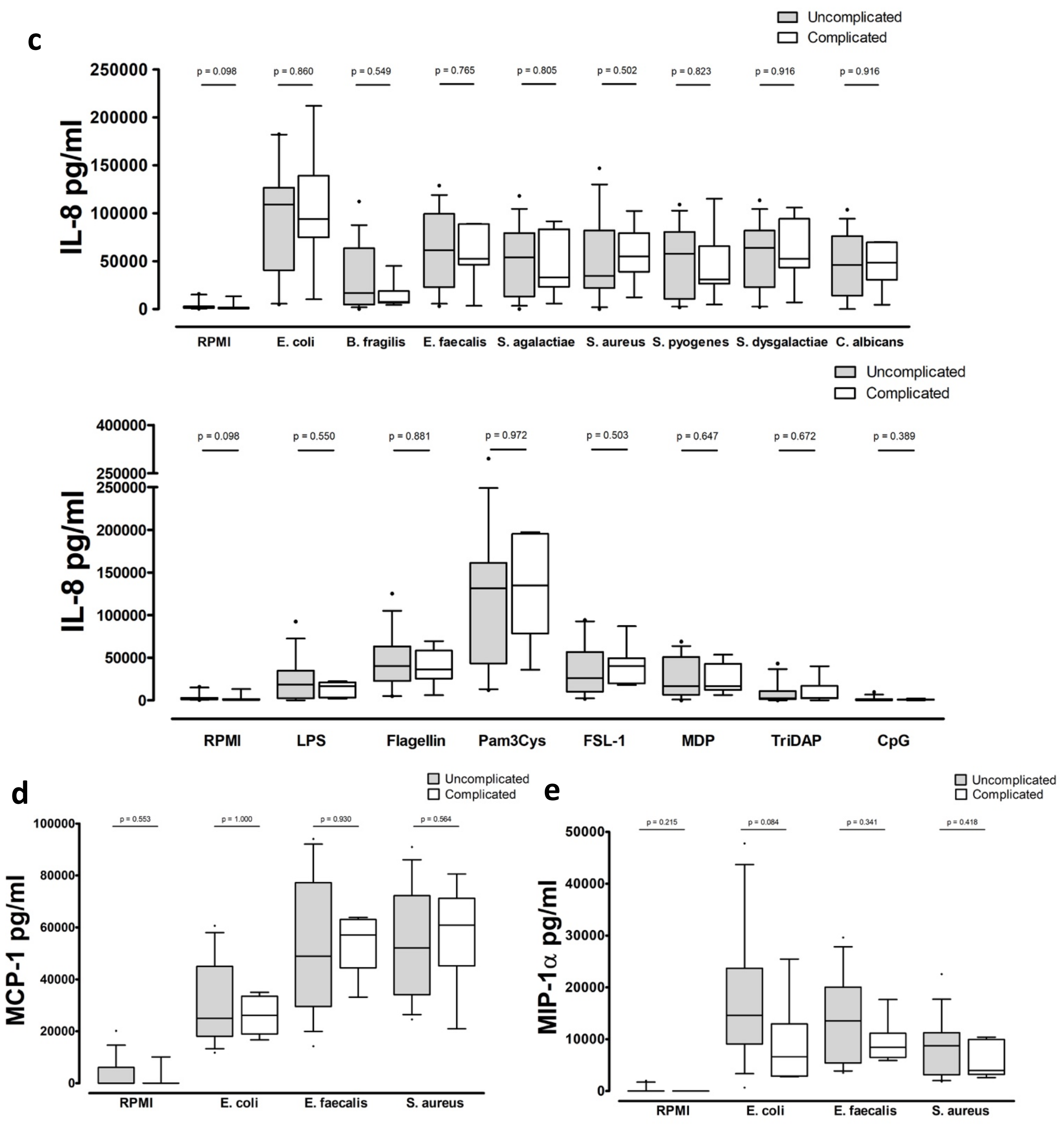
c
Uncomplicated
Complicated
IL-8 pg/ml
250000
200000
150000
100000
50000
0
p = 0.098
p = 0.860
p = 0.549
p = 0.765
p = 0.805
p = 0.502
p = 0.823
p = 0.916
p = 0.916
RPMI
E. coli
B. fragilis
E. faecalis
S. agalactiae
S. aureus
S. pyogenes
S. dysgalactiae
C. albicans
Uncomplicated
Complicated
IL-8 pg/ml
400000
250000
250000
200000
150000
100000
50000
0
p = 0.098
p = 0.550
p = 0.881
p = 0.972
p = 0.503
p = 0.647
p = 0.672
p = 0.389
RPMI
LPS
Flagellin
Pam3Cys
FSL-1
MDP
TriDAP
CpG
d
Uncomplicated
Complicated
MCP-1 pg/ml
100000
80000
60000
40000
20000
0
p = 0.553
p = 1.000
p = 0.930
p = 0.564
RPMI
E. coli
E. faecalis
S. aureus
e
Uncomplicated
Complicated
MIP-1α pg/ml
50000
40000
30000
20000
10000
0
p = 0.215
p = 0.084
p = 0.341
p = 0.418
RPMI
E. coli
E. faecalis
S. aureus

Supplementary Figure S2. Cytokine responses towards commensal bacteria, pathogens and PRR agonists in patients with a history of uncomplicated (n=16) and complicated (n=7) appendicitis. (a) IL-1β responses. (b) IL-6 responses. (c) IL-8 responses. (d) MCP-1 responses. (e) MIP-1α responses. Whiskers indicate 10-90 percentile.

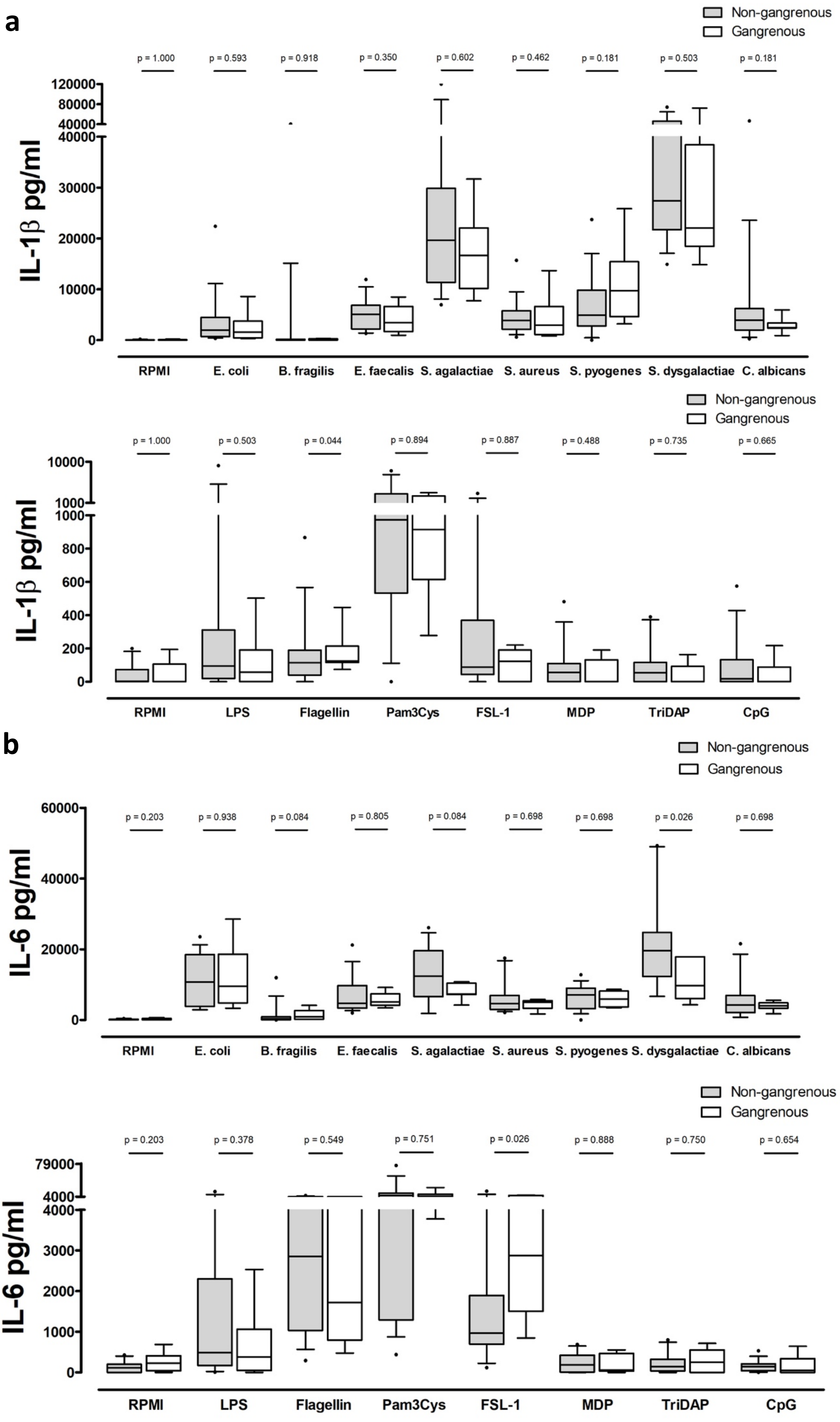
a
Non-gangrenous
Gangrenous
p = 1.000
p = 0.593
p = 0.918
p = 0.350
p = 0.602
p = 0.462
p = 0.181
p = 0.503
p = 0.181
IL-1β pg/ml
120000
80000
40000
40000
30000
20000
10000
0
RPMI
E. coli
B. fragilis
E. faecalis
S. agalactiae
S. aureus
S. pyogenes
S. dysgalactiae
C. albicans
Non-gangrenous
Gangrenous
p = 1.000
p = 0.503
p = 0.044
p = 0.894
p = 0.887
p = 0.488
p = 0.735
p = 0.665
IL-1β pg/ml
10000
1000
1000
800
600
400
200
0
RPMI
LPS
Flagellin
Pam3Cys
FSL-1
MDP
TriDAP
CpG
b
Non-gangrenous
Gangrenous
p = 0.203
p = 0.938
p = 0.084
p = 0.805
p = 0.084
p = 0.698
p = 0.698
p = 0.026
p = 0.698
IL-6 pg/ml
60000
40000
20000
0
RPMI
E. coli
B. fragilis
E. faecalis
S. agalactiae
S. aureus
S. pyogenes
S. dysgalactiae
C. albicans
Non-gangrenous
Gangrenous
p = 0.203
p = 0.378
p = 0.549
p = 0.751
p = 0.026
p = 0.888
p = 0.750
p = 0.654
IL-6 pg/ml
79000
4000
4000
3000
2000
1000
0
RPMI
LPS
Flagellin
Pam3Cys
FSL-1
MDP
TriDAP
CpG

c

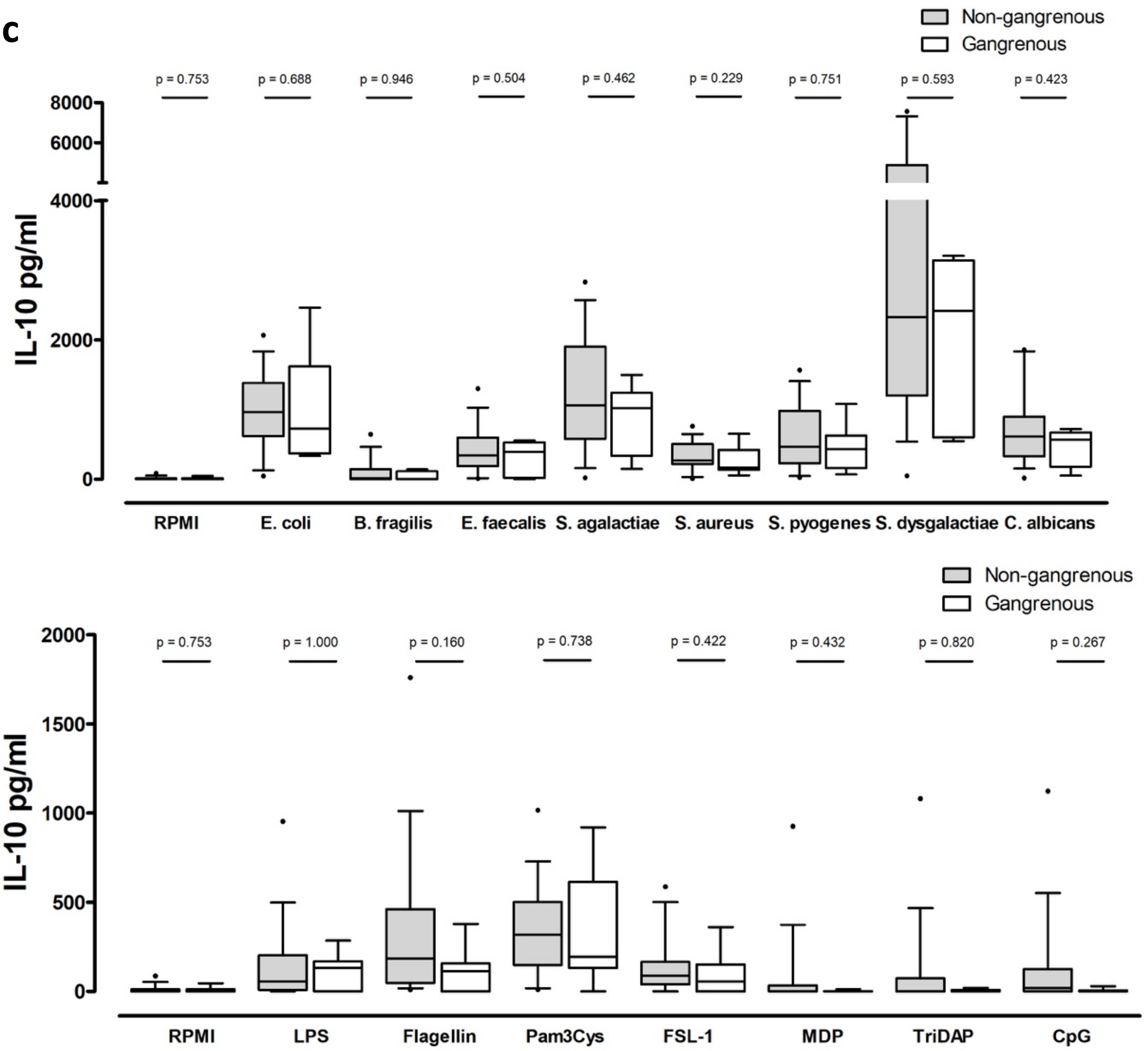
Non-gangrenous
Gangrenous
IL-10 pg/ml
p = 0.753
p = 0.688
p = 0.946
p = 0.504
p = 0.462
p = 0.229
p = 0.751
p = 0.593
p = 0.423
8000
6000
4000
2000
0
RPMI
E. coli
B. fragilis
E. faecalis
S. agalactiae
S. aureus
S. pyogenes
S. dysgalactiae
C. albicans
Non-gangrenous
Gangrenous
IL-10 pg/ml
p = 0.753
p = 1.000
p = 0.160
p = 0.738
p = 0.422
p = 0.432
p = 0.820
p = 0.267
2000
1500
1000
500
0
RPMI
LPS
Flagellin
Pam3Cys
FSL-1
MDP
TriDAP
CpG

d

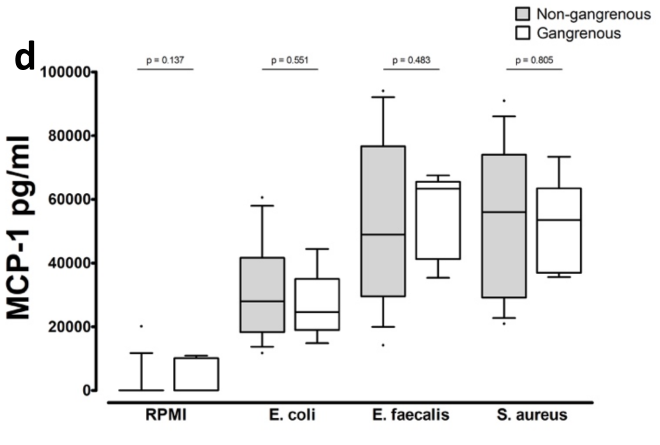
Non-gangrenous
Gangrenous
MCP-1 pg/ml
p = 0.137
p = 0.551
p = 0.483
p = 0.805
100000
80000
60000
40000
20000
0
RPMI
E. coli
E. faecalis
S. aureus

e

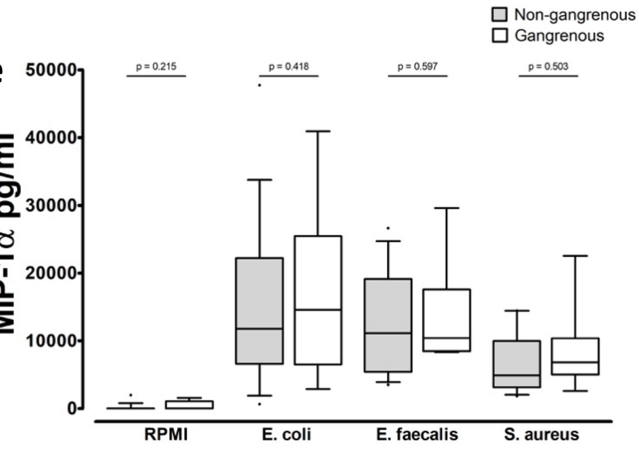
Non-gangrenous
Gangrenous
MIP-1α pg/ml
p = 0.215
p = 0.418
p = 0.597
p = 0.503
50000
40000
30000
20000
10000
0
RPMI
E. coli
E. faecalis
S. aureus

Supplementary Figure S3. Cytokine responses towards commensal bacteria, pathogens and PRR agonists in patients with a history of non-gangrenous (n=16) and gangrenous (n=7) appendicitis. (a) IL-1β responses. (b) IL-6 responses. (c) IL-10 responses. (d) MCP-1 responses. (e) MIP-1α responses. Whiskers indicate 10-90 percentile.
